# Supplementary material for: Effects of passive heat stress and recovery on human cognitive function: An ERP study
Source: PLoS One. 2021 Jul 20;16(7):e0254769. doi: 10.1371/journal.pone.0254769 (PMC8291678; doi:10.1371/journal.pone.0254769)
Supplement: S1 Table — (DOCX) [file pone.0254769.s002.docx]

S1 Table. Mean values for peak amplitude and latency of P300 during Flanker tasks with SD.

|  | Con |  |  |  |  | Incon |  |  |  |  |
| --- | --- | --- | --- | --- | --- | --- | --- | --- | --- | --- |
|  | Fz | Cz | Pz | C3 | C4 | Fz | Cz | Pz | C3 | C4 |
| Amplitude (μV) |  |  |  |  |  |  |  |  |  |  |
| Pre | 8.9 (2.9) | 12.8 (3.6) | 13.2 (4.2) | 10.3 (2.6) | 10.7 (2.9) | 8.5 (3.0) | 12.1 (3.3) | 12.1 (3.7) | 9.8 (2.0) | 10.1 (2.5) |
| Heat | 6.6 (4.0) | 10.4 (3.8) | 11.1 (2.6) | 7.1 (3.4) | 8.1 (2.5) | 5.6 (4.1) | 9.9 (4.5) | 9.6 (3.4) | 5.8 (3.7) | 7.2 (3.6) |
| Recovery | 8.5 (3.6) | 12.0 (4.8) | 12.0 (4.8) | 9.2 (2.7) | 10.0 (3.7) | 8.4 (3.9) | 12.0 (4.9) | 11.9 (4.6) | 8.9 (2.9) | 9.5 (3.3) |
|  |  |  |  |  |  |  |  |  |  |  |
| Latency (ms) |  |  |  |  |  |  |  |  |  |  |
| Pre | 398 (30) | 395 (32) | 381 (40) | 395 (30) | 401 (26) | 408 (42) | 388 (29) | 384 (34) | 396 (30) | 404 (25) |
| Heat | 381 (29) | 373 (30) | 346 (34) | 370 (29) | 375 (27) | 401 (43) | 384 (32) | 356 (31) | 383 (28) | 383 (38) |
| Recovery | 402 (44) | 372 (33) | 364 (40) | 385 (33) | 386 (36) | 416 (49) | 390 (48) | 377 (43) | 408 (40) | 390 (38) |

Con = Congruent stimulus; Incon = Incongruent stimulus
