# Supplementary material for: Effects of passive heat stress and recovery on human cognitive function: An ERP study
Source: PLoS One. 2021 Jul 20;16(7):e0254769. doi: 10.1371/journal.pone.0254769 (PMC8291678; doi:10.1371/journal.pone.0254769)
Supplement: Attachment — Submitted filename: Comments.docx [file pone.0254769.s003.docx]

In this study the authors explore the effects of passive heat stress and recovery on the human cognitive function with Flanker tasks, involving congruent and incongruent stimuli. The results showed the reaction time (RT) was shortened during Heat rather than Pre and Recovery, and the peak amplitudes of the P300 component in ERPs were significantly smaller during Heat than during Pre, suggesting that hyperthermia alters the human cognitive function. However, I would have below comments for authors to improve the work.

1. In the abstract, there was unclear description on cognitive function, such as “… indicating a recuperated cognitive function…”, “These results suggest that hyperthermia alters the human cognitive function…”. Human cognitive function involves multiple factors, such as attention, memory, decision and so on.
2. In the introduction, “Several non-invasive recording methods have been used to investigate the human cognitive function.”, what’s the non-invasive recording methods? Please give more description.
3. In the last paragraph of the introduction, it looks like the section of discussion, please adjust the paragraph and provide more background introduction.
4. “Sixteen subjects (thirteen males and three females; mean age 21.4 ± 1.0 years) participated in this study”，it reflected the mismatch in the factor of gender. Whether the gender difference has an impact on the effects of passive heat stress and recovery on human cognitive function? Please give more explain.
5. How long does the Flanker task last, and how many trials in total does the task consist of for each subject?
6. In the section of EEG recordings, there only were 5 electrodes, and need make sure the quality of EEG data, what is the impedance of the five electrodes across the whole EEG experiment? And provide the detailed information.
7. In the section Discussion, the logic needs to be strengthened, and provide more supporting materials to support the finding “The effectiveness of cooling on impaired cognitive function in hyper-thermic individuals”.
