# Supplementary material for: Effects of passive heat stress and recovery on human cognitive function: An ERP study
Source: PLoS One. 2021 Jul 20;16(7):e0254769. doi: 10.1371/journal.pone.0254769 (PMC8291678; doi:10.1371/journal.pone.0254769)
Supplement: Attachment — Submitted filename: Response to Reviewers0119.docx [file pone.0254769.s004.docx]

**Response to Editorial Office**

This manuscript needs to be significantly improved before it can be accepted for publication in PLoS ONE.

Journal Requirements:

When submitting your revision, we need you to address these additional requirements.

1. Please ensure that your manuscript meets PLOS ONE's style requirements, including those for file naming.

We revised our manuscript meeting PLOS ONE's style requirements.

2. PLOS requires an ORCID iD for the corresponding author in Editorial Manager on papers submitted after December 6th, 2016. Please ensure that you have an ORCID iD and that it is validated in Editorial Manager. To do this, go to ‘Update my Information’ (in the upper left-hand corner of the main menu), and click on the Fetch/Validate link next to the ORCID field. This will take you to the ORCID site and allow you to create a new iD or authenticate a pre-existing iD in Editorial Manager.

We added the ORCiD ID for the corresponding author (Manabu Shibasaki) in the title page.

**Response to Reviewer #1**

Reviewer #1: In this study the authors explore the effects of passive heat stress and recovery on the human cognitive function with Flanker tasks, involving congruent and incongruent stimuli. The results showed the reaction time (RT) was shortened during Heat rather than Pre and Recovery, and the peak amplitudes of the P300 component in ERPs were significantly smaller during Heat than during Pre, suggesting that hyperthermia alters the human cognitive function. However, I would have below comments for authors to improve the work.

1. In the abstract, there was unclear description on cognitive function, such as “… indicating a recuperated cognitive function…”, “These results suggest that hyperthermia alters the human cognitive function…”. Human cognitive function involves multiple factors, such as attention, memory, decision and so on.

We revised these parts (from page 2, line 47 to page 3, line 58):

“However, the peak amplitudes of the P300 component in ERPs, which involved selective attention, expectancy, and memory updating, were significantly smaller during Heat than during Pre, suggesting the impairment of neural activity in cognitive function. Notably, the peak amplitudes of the P300 component were higher during Recovery than during Heat, indicating that the impaired neural activity had recovered after sufficient whole-body cooling. An indicator of the stimulus classification/evaluation time (peak latency of P300) and the RT were shortened during Heat stress, but such shortening was not noted after whole-body cooling. These results suggest that hyperthermia affects the human cognitive function, reflected by the peak amplitude and latency of the P300 component in ERPs during the Flanker tasks,”

2. In the introduction, “Several non-invasive recording methods have been used to investigate the human cognitive function.”, what’s the non-invasive recording methods? Please give more description.

We revised this part (page 4, lines 93-96):

“Several non-invasive recording methods have been used to investigate the human cognitive function, such as functional magnetic resonance imaging (fMRI), functional near-infrared spectroscopy (fNIRS), and transcranial magnetic stimulation (TMS).”

3. In the last paragraph of the introduction, it looks like the section of discussion, please adjust the paragraph and provide more background introduction.

We revised this part, as suggested (page 6, lines 129-145):

“Thus, we chose “visual” Flanker tasks, which were used to examine the neural systems that resolve the conflict among response options [12]. In these tasks, a central target stimulus is presented simultaneously with distractor stimuli (flankers) and subjects are requested to respond according to the target and ignore the flankers. In general, the reaction time (RT) is shorter with Congruent stimuli than Incongruent stimuli [13, 14], indicating different levels of task difficulty [13, 14]. The second involves recovery after heat stress. As mentioned above, a short period of whole-body cooling after heat stress and face/head cooling during heat stress did not lead to full recovery of the peak amplitude of P300 [9, 10]. In addition, after a thorough literature research, we failed to identify any study examining the recovery process after heat stress by P300. Providing scientific evidence to support recovery from hyperthermia might be important to develop a methodology in our daily life and sports activities. Thus, we should examine whether sufficient whole-body cooling recovers the cognitive function reflected by the P300 component in ERPs.”

4. “Sixteen subjects (thirteen males and three females; mean age 21.4 ± 1.0 years) participated in this study”, it reflected the mismatch in the factor of gender. Whether the gender difference has an impact on the effects of passive heat stress and recovery on human cognitive function? Please give more explain.

Thank you for this suggestion. We added the explanation in limitations of the present study (page 20, lines 466-474):

“Finally, the present study recorded the data from thirteen males and three females, but did not investigate gender differences in the effects of passive heat stress and recovery on the human cognitive function. It is well-known that there are gender differences in thermoregulatory responses [31, 32]. In this study, we controlled the subjects’ internal temperature with a water-perfused suit, which minimized individuals’ thermoregulatory capacity. Therefore, any gender difference in thermoregulatory responses might affect the cognitive function during the process of recovery from hyperthermia. Further studies are needed to clarify this.”

5. How long does the Flanker task last, and how many trials in total does the task consist of for each subject?

We added the explanation (from page 8, line 196 to page 9, line 203):

“A run comprised 160 stimuli (i.e., 8 min), which included 40 stimuli for the left congruent arrowhead, 40 stimuli for the right congruent arrowhead, 40 stimuli for the left incongruent arrowhead, and 40 stimuli for the right incongruent arrowhead. The probability of all stimuli was equal, being presented in a random order. Inter-stimulus interval was fixed. Pre, Heat, and Recovery sessions included 160 stimuli, respectively. In a practice run, subjects were instructed to perform the Flanker tasks with 40 stimuli before recording the Pre session.”

6. In the section of EEG recordings, there only were 5 electrodes, and need make sure the quality of EEG data, what is the impedance of the five electrodes across the whole EEG experiment? And provide the detailed information.

We added the explanation (page 10, lines 236-239):

“All electrodes were detached after ERP recordings in Pre and Heat sessions, respectively, to avoid the effect of sweat on the EEG paste. Just before ERP recordings in Heat and Recovery sessions, all electrodes were again attached.”

7. In the section Discussion, the logic needs to be strengthened, and provide more supporting materials to support the finding “The effectiveness of cooling on impaired cognitive function in hyper-thermic individuals”.

Thank you for this constructive comment. We revised this section (from page 17, line 391 to page 19, line 440).

**Response to Reviewer #2**

Concentrating on the effect of hyperthermia on human cognition, the authors conducted the Flanker tasks to investigate the differences of ERP and behaviors across different conditions. They found the varied measurements across multiple factors, although these results seem sound, concerns should be resolved by authors before considering for publication.

1. Although the authors mentioned the aim of their study, more information should be given for their novelty and purpose for hyperthermia and related ERP study.

Thank you for this suggestion. We revised the Introduction section (page 6, lines 129-145):

“Thus, we chose “visual” Flanker tasks, which were used to examine the neural systems that resolve the conflict among response options [12]. In these tasks, a central target stimulus is presented simultaneously with distractor stimuli (flankers) and subjects are requested to respond according to the target and ignore the flankers. In general, the reaction time (RT) is shorter with Congruent stimuli than Incongruent stimuli [13, 14], indicating different levels of task difficulty [13, 14]. The second involves recovery after heat stress. As mentioned above, a short period of whole-body cooling after heat stress and face/head cooling during heat stress did not lead to full recovery of the peak amplitude of P300 [9, 10]. In addition, after a thorough literature research, we failed to identify any study examining the recovery process after heat stress by P300. Providing scientific evidence to support recovery from hyperthermia might be important to develop a methodology in our daily life and sports activities. Thus, we should examine whether sufficient whole-body cooling recovers the cognitive function reflected by the P300 component in ERPs.”

2. Have the authors considered the gender effect on their results, since only three females were included in their study.

Thank you for this suggestion. Reviewer #1 also pointed out the same problem. We added the explanation in limitations of the present study (page 20, lines 466-474):

“Finally, the present study recorded the data from thirteen males and three females, but did not investigate gender differences in the effects of passive heat stress and recovery on the human cognitive function. It is well-known that there are gender differences in thermoregulatory responses [31, 32]. In this study, we controlled the subjects’ internal temperature with a water-perfused suit, which minimized individuals’ thermoregulatory capacity. Therefore, any gender difference in thermoregulatory responses might affect the cognitive function during the process of recovery from hyperthermia. Further studies are needed to clarify this.”

3. Since the authors said the ’incompatible’ setting was used in Flanker task, for congruent and incongruent conditions, how the subjects responded to these varied stimuli.

We added the explanation (page 8, lines 188-194):

“For example, when the central arrowhead was directed to the left, the subjects were instructed to press a button with their left thumb. However, after being trained in the compatible setting, subjects were requested to respond in a direction opposite to the target arrowhead, called an ‘incompatible’ setting. For example, when the central arrowhead was directed to the left, the subjects were instructed to press a button with their right thumb.”

4. Only five channels were recorded, if the authors tried any referencing techniques during their data preprocessing.

Reviewer #1 also pointed out the same problem. We added the explanation (page 10, lines 230-231):

“Each scalp electrode was referenced to linked earlobes, which were mathematically calculated and averaged for reference.”

5. For statistics, how did the authors perform the multiple comparison correction for ERP and behavior measurements?

We performed the Bonferroni post-hoc multiple-comparison to identify the specific differences among sessions, but did not adjust the comparison correction. We added the explanation (from page 11, line 267 to page 12, line 277):

“For all repeated-measures factors, we tested whether Mauchly’s sphericity assumption was violated. If the result of Mauchly’s test was significant and the assumption of sphericity was violated, Greenhouse-Geisser adjustment was used to correct sphericity by altering the degrees of freedom using a correction coefficient epsilon. When a significant effect of Session was identified, the Bonferroni post-hoc multiple-comparison was adjusted to identify the specific differences among sessions. When a significant effect of Stimulus was identified, the post-hoc paired-t-test was adjusted to identify the specific difference between Congruent and Incongruent stimuli. We did not adjust the multiple comparison correction for all data.”

6. If it is necessary to present the results for SD of RT when introducing their results.

Thank you for this helpful comment. We added the explanation on the results for SD of RT in many parts of Discussion sections.

7. How could the authors explain the confusing results of shorter RT and latency but decreased P300 amplitude for HEAT condition.

We revised the Discussion section (from page 18, line 424 to page 19, line 440):

“We considered that acceleration of RT and the latency of P300 and small response variability were related to specific effective connectivity in neural networks with an increasing internal temperature, which differs from the theory of structural connectivity [see a review, 27]. Kao et al. [28] recently reported that the peak amplitude of P300 in Flanker tasks was reduced, and that the peak latencies of P300 and RT were shortened after high-intensity interval training on a treadmill. Since the modulation of P300 was very similar to our findings, their data might be related to the effects of an increasing body temperature as well as the exercise of running on a treadmill. We hypothesized that the effects of an increasing body temperature on P300 differed between the amplitude and latency, paradoxically. Indeed, a study using multi-channel EEG recording showed that the resting-state network topology was significantly correlated with the amplitude of P300, but not the latency [29]. This study suggests that the amplitude and latency of P300 is related to different neural mechanisms, networks, and/or functional connectivity in the whole brain.”

8. If it is suitable to using the Flanker task to index the task difficulty, the authors should give some explanation.

We added the Introduction section to explain the task difficulty in the Flanker task (page 6, lines 129-135):

“Thus, we chose “visual” Flanker tasks, which were used to examine the neural systems that resolve the conflict among response options [12]. In these tasks, a central target stimulus is presented simultaneously with distractor stimuli (flankers) and subjects are requested to respond according to the target and ignore the flankers. In general, the reaction time (RT) is shorter with Congruent stimuli than Incongruent stimuli [13, 14], indicating different levels of task difficulty [13, 14].”

9. As depicted, more electrodes would be needed to clarify the brain mechanism accounting for effect of hyperthermia on P300, since our brain works as a large-scale complex network, and concentrating on P300 network, many related works have been implemented, such as

> F. Li, et al., Scientific Reports, 2015, 5: 15129; W. Peng, et al., PLoS One, 2012, 7(4):e34163

Thank you for this suggestion. We added the explanation in Discussion (from page 18, line 433 to page 19, line 440):

“We hypothesized that the effects of an increasing body temperature on P300 differed between the amplitude and latency, paradoxically. Indeed, a study using multi-channel EEG recording showed that the resting-state network topology was significantly correlated with the amplitude of P300, but not the latency [29]. This study suggests that the amplitude and latency of P300 is related to different neural mechanisms, networks, and/or functional connectivity in the whole brain.”

Limitations of the present study (from page 19, line 458 to page 20, line 462):

“Our ERP methods involving recordings from five electrodes did not directly clarify the brain regions associated with the reduced amplitude of P300 or the accelerated latency of P300. Therefore, multi-channel ERP recording would be needed to investigate the generator mechanisms, neural networks, and functional connectivity of P300.”
