# Supplementary material for: Effects of passive heat stress and recovery on human cognitive function: An ERP study
Source: PLoS One. 2021 Jul 20;16(7):e0254769. doi: 10.1371/journal.pone.0254769 (PMC8291678; doi:10.1371/journal.pone.0254769)
Supplement: Attachment — Submitted filename: Response to ReviewersPONE-D-20-33346R1.docx [file pone.0254769.s005.docx]

**Response to Reviewer #3**

The manuscript reports analyses of ERP data before, during, and after participants underwent a heat stress challenge paradigm. These data must have been demanding to acquire and the authors are to be congratulated on this. I have a few points I would like them to respond to, a few substantive issues followed by more minor points:

1. The changes to cognitive function during heat stress and recovery are framed as impairments in the discussion, but the actual task performance, in terms of RT and consistency, is improved, while accuracy appears similar across conditions (perhaps a ceiling effect?). Yes the P300 was smaller in amplitude, but I don’t think that is evidence of impairment in of itself. This needs some attention in the Discussion.

We added the explanation in the Discussion (page 19, lines 437-455):

“The interpretation of these results requires special attention. We considered that acceleration of RT and the latency of P300 and smaller SD of RT (response variability) were related to specific effective connectivity in neural networks with an increasing internal temperature, which differs from the theory of structural connectivity [see a review, 30]. Kao et al. [31] recently reported that the peak amplitude of P300 in Flanker tasks was reduced, and that the peak latencies of P300 and RT were shortened after high-intensity interval training on a treadmill. Since the modulation of P300 and RT were very similar to our findings, their data might be related to the effects of an increasing body temperature as well as the exercise of running on a treadmill. We hypothesized that the effects of an increasing body temperature on P300 differed between the amplitude and latency, paradoxically. Indeed, a study using multi-channel EEG recording showed that the resting-state network topology was significantly correlated with the amplitude of P300, but not the latency [32]. This study suggests that the amplitude and latency of P300 is related to different neural mechanisms, networks, and/or functional connectivity in the whole brain. In future studies, these parameters should be analyzed during passive heat stress.”

2. I don’t see any mention of artefact correction or rejection – were all trials used? If EEG data quality differed between conditions, that could plausibly drive effects in component amplitudes – are you able to assess how likely that is to be a problem?

We added the explanation in Materials and Methods section (page 10, lines 235-237):

“Artifacts or noise caused by blinking or sweating were excluded on-line. If the number averaged was less than 35 for each stimulus, additional trials were performed.”

3. Related to the above point, you mention that electrodes were detached between conditions to minimise sweat artefacts. Can you reassure me that this dealt with this, as this does seem like a potentially important issue?

Thank you for constructive comment. We added the discussion on this issue (page 17, lines 390-399):

“In addition, the reduced amplitude of P300 might be related to electrical noise due to sweating during passive heat stress. However, as mentioned above, to avoid the effect of sweat on the EEG paste, we reattached all electrodes just before ERP recordings in Heat and Recovery sessions. Our previous study using the same procedure as in the present study showed that the amplitudes of some components in somatosensory evoked potentials (SEPs) reduced during passive heat stress, but other components did not [22, 23]. If the presence of sweat influences the SEP waveforms, all SEP components should be distorted. Therefore, we considered that sweat had a reduced effect on the amplitude of P300 in the present study.”

4. The theoretical relevance of changes to variability of RTs is not discussed.

We added the discussion (from page 18, line 430 to page 19, line 455).

Minor issues

1. Breaking the introduction into more paragraphs might make it a bit more reader friendly.

We revised the introduction section.

2. Page 7 – ‘The man body mass and height…’ – Should ‘man’ read ‘mean’?

Revised (page 7, line 155):

“The mean body mass and height of the subjects were 71.7 ± 11.3 kg, and 170.5 ± 6.3 cm, respectively.”

3. Page 8 – ‘the 2nd ERP was recorded after…’ – I would change to ‘ERP session was’ or ‘ERPs were’. Likewise for ‘the 3rd ERP

Revised (page 8, line 175):

“ERPs were recorded after the external ear canal temperature”

Revised (page 8, line 180):

“ERPs were recorded (Recovery session) (Fig 1A).”

4. Page 12 – ‘Significance was set at p < 0.05.’ – ‘significance’ should read ‘alpha’

Revised (page 12, line 281):

“All statistical analyses were conducted with α = 0.05.”
